# Characterization of an Isolate of Citrus Concave Gum-Associated Virus from Apples in China and Development of an RT-RPA Assay for the Rapid Detection of the Virus

**DOI:** 10.3390/plants10112239

**Published:** 2021-10-20

**Authors:** Zhen Liu, Zhenfei Dong, Binhui Zhan, Shifang Li

**Affiliations:** 1 State Key Laboratory for Biology of Plant Diseases and Insect Pests, Institute of Plant Protection, Chinese Academy of Agricultural Sciences, Beijing 100193, China; liuzhencaas@163.com (Z.L.); dongzhenfeiqaz@163.com (Z.D.); 2 Department of Fruit Science, College of Horticulture, China Agricultural University, Beijing 100193, China

**Keywords:** citrus concave gum-associated virus, apple virus, coguvirus, RT-RPA

## Abstract

Apple (*Malus domestica*) fruits exhibiting bright stripe symptoms were identified in Weihai City, Shandong Province, China. To investigate the virome in the apple samples, the method of high throughput sequencing (HTS) was used to identify the viruses. It was found that the sequence of citrus concave gum-associated virus (CCGaV) was involved in the apple transcriptome dataset. The full-length genome of the CCGaV-Weihai isolate contained two segments, the RNA1 was 6674 nt in size containing a conserved RNA-dependent RNA polymerase (RdRp), and the RNA2 was ambisense, 2706 nt in length, encoding a movement protein (MP) and a coat protein (CP). Sequence alignment and phylogenetic analyses indicated that CCGaV-Weihai was more closely related to CCGaV-H2799 isolated from the apple host in the United States and distantly related to CCGaV-CGW2 from *Citrus sinensis* in Italy, indicating a possibly geographical and host differentiation of CCGaV isolates. This was the first identification and characterization of CCGaV infecting apples in China. Additionally, a rapid and sensitive reverse transcription recombinase polymerase amplification (RT-RPA) assay technique was established for CCGaV detection in apple plants. The RT-RPA of CCGaV was not affected by other common viruses in apple plants and is about 10-fold more sensitive than the conventional reverse transcription polymerase chain reaction method, which can be used in large-scale testing.

## 1. Introduction

Apple (*Malus domestica*) is an economically important fruit crop in many areas of the world. China was the largest producer of apples in 2019, with approximately 38.1% of the world cultivating area and 51.1% of total crop production (FAOSTAT, http://faostat.fao.org, accessed on 30 August 2021). The yield and quality of apples have been affected by the incidence and severity of various diseases, and viral diseases are one of the most common constraints. Dozens of viruses or viroids have been reported to infect apple trees worldwide, leading to the symptoms of necrosis, mosaic and apple decline. Globally, apple decline has increased in incidence, and several pathogens have been proposed to be associated with this disease, including apple chlorotic leafspot virus (ACLSV) and apple stem grooving virus (ASGV), while no one or group of pathogens have been totally determined [1,2].

Negative-stranded RNA (nsRNA) viruses are dangerous pathogens which pose great threats to humans, animals and plants. Recently, a novel genus named *Coguvirus* has been proposed in the family *Phenuiviridae* and order *Bunyavirales* [3], which contains two bipartite nsRNA viruses: citrus concave gum-associated virus (CCGaV) [4,5,6] and citrus virus A (CiVA) [7]. In addition, nsRNA viruses with similar characteristics to members belonging to the genus *Coguvirus* identified in watermelon and grapevine are pending classification [8,9,10,11]. Citrus concave gum-associated virus (CCGaV) is one of the coguviruses infecting citrus [4] and apple plants [5,6] which is proposed to be associated with graft-transmissible citrus concave gum-blind pocket disease [4]. The genome of CCGaV contains two segments, RNA1 encoding the viral RNA-dependent RNA polymerase (RdRp), and ambisense RNA2 encoding putative movement protein (MP) and nucleocapsid protein (CP) [4]. CCGaV RNA1 and RNA2 share almost identical nucleotide sequences at the 5′ and 3′ terminal sequence, and the 5′-terminal motif ACACA and 3′-terminal motif UGUGU are conserved in the members of the family *Phenuiviridae* [4]. The 5′ and 3′ ends of each genomic RNA are complementary, forming a panhandle structure [4]. The MP and CP open reading frames were separated by a long AU-rich intergenic region (IR) with a hairpin conformation [4]. Recently, CCGaV was identified in apples in Brazil and thr United States and in apple trees originally from Australia, France, Italy and Spain maintained in a collection field in USA [5,6], implying that CCGaV is globally distributed in apples, which is reasonable considering the global trade in propagative material.

The characteristics of perennation, grafting propagation and the need for pruning make it difficult to manage fruit trees once they are infected by viruses or viroids, resulting in potential losses [12]. The use of virus-free propagative material has been and is an effective method of controlling viral fruit tree diseases [13]. The detection of fruit tree viruses is challenging compared with annual crops, due to the low titer, uneven distribution in the plants, the occurrence of mixed-infection in single tree and symptomlessness or suspicious symptoms during different seasons [12]. Recently, many methods have been used for detection of fruit tree viruses, including enzyme-linked immunosorbent assays (ELISA), reverse transcription polymerase chain reactions (RT-PCR) and high throughput sequencing (HTS). However, each method has its limitations and is not suitable for testing all fruit tree viruses. ELISA, Western blots and other immunological methods are restricted by the preparation of highly effective antibodies. Nucleic acid-based RT-PCR or real-time RT-PCR are time-consuming and require expensive equipment. HTS is suitable for detecting viromes in samples and requires expert bioinformaticians to perform analysis. Therefore, it is necessary to establish a rapid, simple and effective method for the detection of low-titer fruit tree viruses to help control virus transmission.

An alternative molecular technology, recombinase polymerase amplification (RPA), can isothermally amplify nucleic acid and was developed for detection of different pathogens [14]. The RPA process utilizes recombinases which can bind to single-stranded nucleic acid backbones and stimulate the resulting protein-DNA complex to search for homologous sequences [14,15]. Once the homology is located, the oligonucleotide is paired to its complement permitting a polymerase to begin synthesis from the 3′ end [14]. Two opposing primers designed for a target, in a manner similar to that for PCR, permit the establishment of exponential amplification from a few target copies in less than 30 min with an acceptable level of sensitivity and specificity. The advantage of this method is that the reaction runs at a constant temperature of about 37–42 °C without the need for sophisticated thermal cyclers [16], which is suitable for the rapid detection of fruit tree viruses, such as little cherry virus 2 [17], plum pox virus [18], apple stem pitting virus (ASPV) [19,20] and apple necrotic mosaic virus (ApNMV) [20].

In this study, an isolate of apple-infecting CCGaV (CCGaV-Weihai) was obtained, and bioinformatic analyses of CCGaV-Weihai were conducted to reveal its relationship with other CCGaV isolates. Moreover, an RT-RPA assay was established for the rapid, sensitive and effective detection of CCGaV in apple trees.

## 2. Results

### 2.1. Identification of Virome Using High Throughput Sequencing

**Figure 1:**
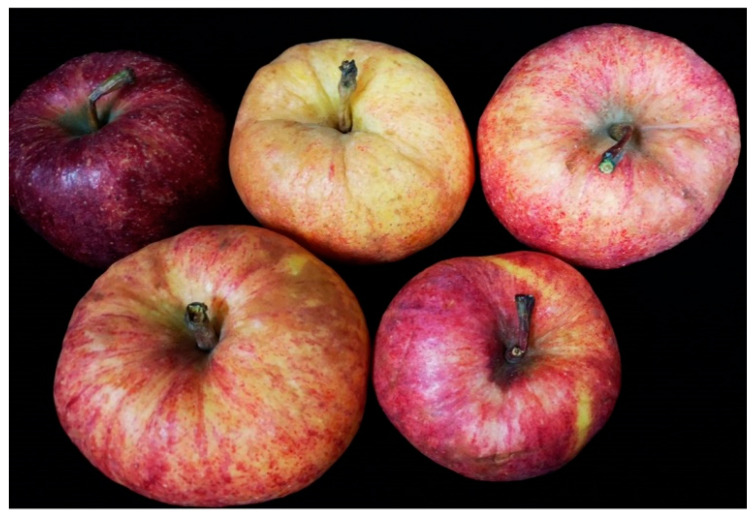
Symptoms of apple fruits showing bright stripe in Weihai City, Shandong Province, China.

In October 2020, apple fruits showing obvious bright stripe symptoms were observed in Weihai City, Shandong Province, China. These fruits were collected and photographed (Figure 1). To identify the viruses in the apple samples, total RNAs were isolated from the peels of 13 apple fruits from different trees and the RNAs were mixed as a single sample which was subjected to HTS. For the HTS, a total of 74,703,526 raw reads were produced and 71,342,876 clean reads were obtained after adapter, quality and length trimming. After filtering the reads mapping to the apple genome, the unmapped reads (6.05% with 4,319,214 reads) were de novo assembled into contigs with Trinity software 9 (Cambridge MA, USA). The results of BLASTn, using the obtained contigs as queries, suggested that six viruses and two viroids exist in the apple transcriptome, i.e., apple chlorotic leaf spot virus (ACLSV), apple stem grooving virus (ASGV), ASPV, ApNMV, CCGaV, apple rubbery wood virus 1 (ARWV1), apple hammerhead viroid (AHVd) and apple scar skin viroid (ASSVd). Viral reads of the six viruses and two viroids are listed in Table S1. In addition, the presence of the six viruses and two viroids were also confirmed by RT-PCR using specific primers (listed in Table S2) in the mixed sample used for HTS (Figure S1). Furthermore, the individual detection of viruses and viroids in 13 samples was conducted, which showed that all samples had mixed infections. The detailed detection results are listed in Table S3. Among them, CCGaV is the first report in China. One larger contig of 6661 nt was annotated as CCGaV RNA1 and two different contigs of 1373 nt and 1297 nt were annotated as CCGaV RNA2 with high-sequence identities. An RT-PCR using primer pair CCGaV-5F/3R validated the presence of CCGaV in 7 out of 13 apple samples.

### 2.2. Full-Length Genomic Sequence and Characterization of CCGaV

RT-PCR amplification with the specific primer pairs (Table S2) and sequencing of the fragments confirmed the presence of CCGaV contigs and determined the viral sequences. In addition, the 5′- and 3′-terminal sequences of CCGaV RNA1 and RNA2 were amplified and sequenced with 5′- and 3′-RACE system (primers listed in Table S2). The sequences of several fragments covering the whole genome amplified from one apple fruit were assembled with DNAMAN software, resulting in the complete CCGaV genomic RNAs. This isolate was named the CCGaV-Weihai isolate.

**Figure 2:**
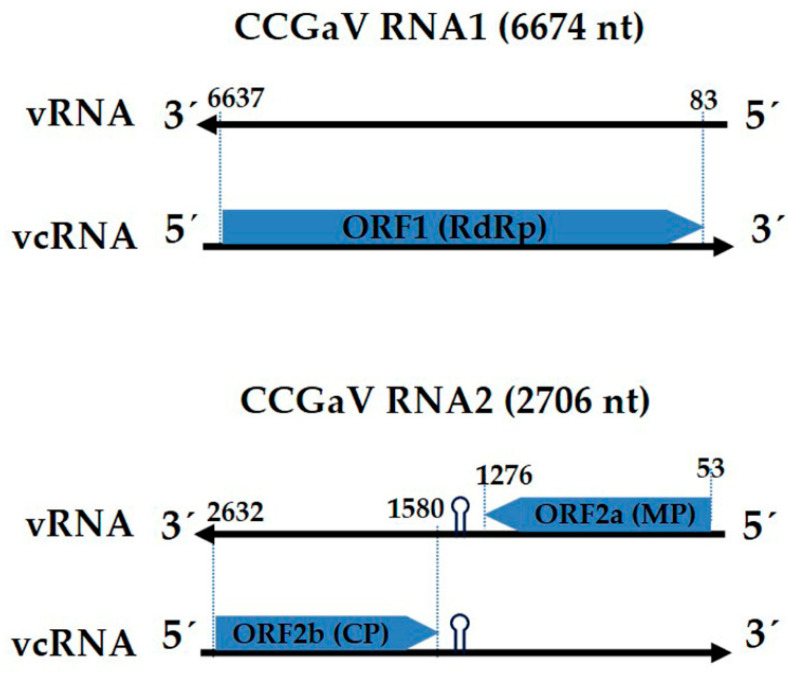
Schematic representation of the genome organization of citrus concave gum-associated virus (CCGaV) RNA1 and RNA2.

The whole genome of the CCGaV-Weihai isolate contained two RNA segments, RNA1 (NCBI GenBank No. MZ926713, accessed on 30 August 2021) with 6674 nt and RNA2 (NCBI GenBank No. MZ926714) with 2706 nt in length (Figure 2). The 5′-termini of the RNA1 and RNA2 from CCGaV-Weihai isolate contained the motif ACACA, and the 3′-termini contained the motif UGUGU, which are conserved in the members of the family *Phenuiviridae*. The RNA1 contained, in the viral complementary (vc) strand, one open reading frame (ORF) which started at position 6637 nt and ended at position 83 nt with a TGA stop codon encoding RdRp of 2184 amino acids (aa) and an estimated molecular weight of −250 kDa. The RdRp of the CCGaV-Weihai isolate was aligned with RdRps of other CCGaV isolates, indicating that the CCGaV-Weihai isolate shared the highest identity with CCGaV-H2799 (NCBI GenBank No. QSC42549) of 99.91% and subsequently with CCGaV-Gala (NCBI GenBank No. QDK54398) of 99.82%, CCGaV-Mishima (NCBI GenBank No. QDK54399) of 99.77%, CCGaV-FT159 (NCBI GenBank No. QTH26260) of 98.99%, CCGaV-AC1 (NCBI GenBank No. AXR98526) of 98.81%, CCGaV-LR3 (NCBI GenBank No. AYN78565) of 97.39% and CCGaV-CGW2 (NCBI GenBank No. YP_009422199) of 97.30% (Table S4). The RNA2 contained two ORFs in an opposite orientation, which were separated by an AU-rich intergenic region (IR) forming a long hairpin conformation (spanning position 1335 to 1462). The highly structured element containing the CUCUGCU motif has been proposed by other CCGaV isolates and several ambisense phleboviruses, which could act as a transcription termination signal (TTS) during viral genome transcription [4]. The ORF2a spanning 53 nt to 1276 nt in CCGaV-Weihai RNA2 encoded a putative MP of 407 aa of 45.8 kDa. ORF2b, contained in the vc strand of RNA2 spanning position 2632 to 1580, encoded a putative CP of 350 aa of 39.1 kDa.

**Figure 3:**
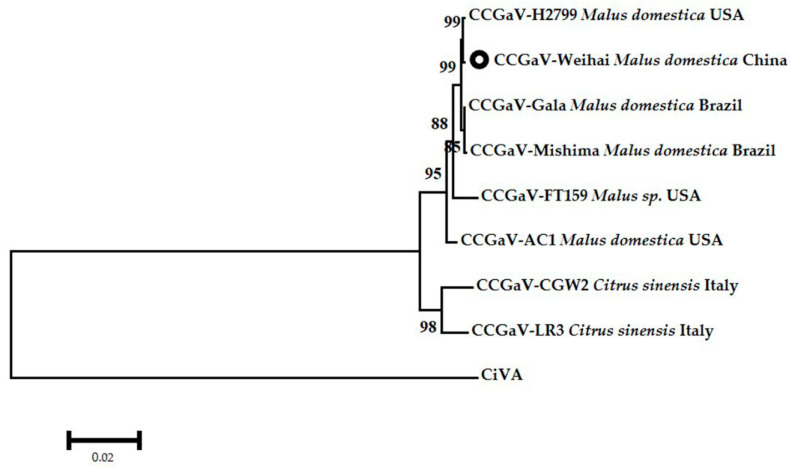
Phylogenetic relationship of CCGaV-Weihai and other isolates of CCGaV from different countries and hosts, based on RdRp amino acid sequences. The phylogenetic tree was constructed using the maximum-likelihood method in MEGA 7.0, with a bootstrap of 1000 replicates. The black circle indicated the CCGaV-Weihai isolate from *Malus domestica* in China.

In addition, multiple alignments of RdRp amino acid sequences of different CCGaV isolates were performed using ClustalW (Heidelberg, Germany)), and then the phylogenetic trees were constructed using the maximum-likelihood method with a bootstrap of 1000 replicates in MEGA 7.0(Philadelphia, USA). The grouping indicated that eight CCGaV isolates differentiated from Citrus virus A (CiVA) which is another nsRNA virus in the *Coguvirus* genus. Within the CCGaV cluster, CCGaV-Weihai is more closely related with CCGaV-H2799, which was isolated from *Malus domestica* in the United States, and is distantly related to CCGaV-CGW2 from *Citrus sinensis* in Italy. The isolates from *Malus domestica* in Brazil are in one subgroup close to the isolates from the host of *Malus domestica* or *Malus* sp. in the United States and China, while the CCGaV-LR3 and CCGaV-CGW2 isolates isolated from *Citrus sinensis* in Italy are in another subgroup belonging to a different clade with respect to the other six CCGaV isolates (Figure 3).

**Figure 4:**
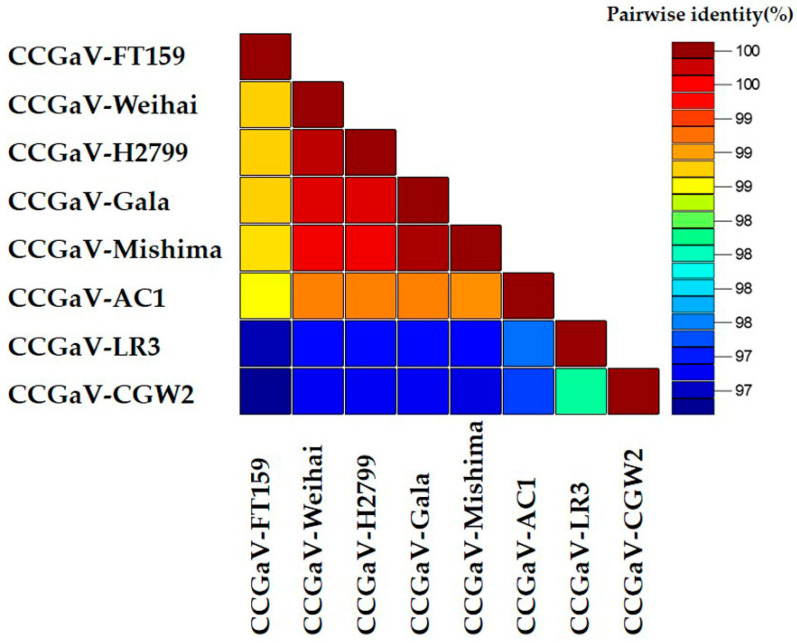
Pairwise identity plot of RdRps of different CCGaV isolates aligned by MAFFT and displayed by SDT software.

To further determine the relationship between different CCGaV isolates, the pairwise alignments of CCGaV RdRps were conducted using the MAFFT program. The SDT software (Cape Town, South Africa) was used to display the pairwise identity scores. The color-coded matrix plot showed that the pairwise identities can be divided into two groups (one group was isolated from *Citrus sinensis* in Italy and the other was isolated from *Malus domestica* or *Malus* sp. in the American region and China), which are consistent with the different hosts and geographical locations of CCGaV isolates (Figure 4 and Table S5).

### 2.3. Establishment of a RT-RPA Assay for CCGaV Detection

In order to detect CCGaV conveniently and quickly, we established an RT-RPA method. Two pairs of RT-RPA primers (Table S2) based on the genomic segment RNA1 were designed for the RT-RPA assay. RPA-F1/R1 and RPA-F2/R2 were predicted to produce amplification products of 198 bp and 174 bp, respectively. The reaction condition of RPA was set at 37 °C for 30 min. The results showed that the specific amplification product of RPA-F1/R1 was obtained from CCGaV-infected apple fruit showing a clear band in the agarose gel electrophoresis assay, and no amplification products were obtained from non CCGaV-infected apple plants (which did contain the ACLSV, ASGV, ASPV and ApNMV viruses) or non-template control (NTC) (Figure S1). The amplicon obtained with RPA-F1/R1 was purified and directly sequenced. Sequence alignment of the specific amplicon showed 100% similarity with the corresponding CCGaV sequence, spanning position 5001 to 5198. The amplification product by RPA-F2/R2 was nonspecific, with smaller products in non CCGaV-infected apple plants and NTC (Figure S2). The primer pair RPA-F1/R1 was selected for further study.

**Figure 5:**
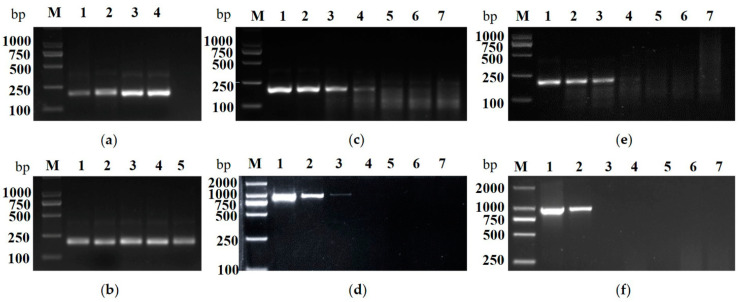
Establishment of RT-RPA assay for CCGaV detection. (**a**) Optimization of RT-RPA reaction temperature. M, Trans2K DNA marker; lane 1–4: 36 °C, 37 °C, 38 °C and 39 °C. (**b**) Optimization of RT-RPA reaction time. M, Trans2K DNA marker; lane 1–5: 10 min, 20 min, 30 min, 40 min and 50 min. Sensitivity comparison of RT-RPA (**c**) and RT-PCR (**d**) using diluted cDNA. Lane M, Trans2K DNA marker; lane 1–5, a series of dilutions of cDNA from 1 μg total RNA from CCGaV-infected apple fruit including 10^0^, 10^−1^, 10^−2^, 10^−3^ and 10^−4^; lane 6, non-template control (NTC); lane 7, non CCGaV-infected apple plant. Sensitivity comparison of RT-RPA (**e**) and RT-PCR (**f**) using diluted total RNA. Lane M, Trans2K DNA marker; lane 1–5, a series of dilutions of 1 μg total RNA including 10^0^, 10^−1^, 10^−2^, 10^−3^ and 10^−4^ from CCGaV-infected apple fruit; lane 6, NTC; lane 7, the non CCGaV-infected apple plant.

To optimize the reaction condition of the RT-RPA assay, a series of reaction temperature (36, 37, 38, 39 °C) were tested (Figure 5a). The temperatures of 38 °C and 39 °C were the better choices. A series of reaction time (10, 20, 30, 40, 50 min) at 38 °C were further investigated and 30 min was the shortest and most suitable time (Figure 5b). Taken together, the optimal reaction condition was set at 38 °C for 30 min. To evaluate the sensitivity of the established RT-RPA assay, a series of dilutions of CCGaV-infected apple plant cDNA (transcripted from 1.0 × 10^3^ ng total RNA) with water, including 10^0^, 10^−1^, 10^−2^, 10^−3^ and 10^−4^, were prepared and amplified by RT-RPA and RT-PCR, respectively. The RT-RPA was conducted as described above, and the RT-PCR was performed with CCGaV-5F/3R. The cDNA from the non-CCGaV-infected apple plant and NTC did not produce any reaction with RT-RPA or RT-PCR. The RT-RPA method could detect CCGaV-infected transcripts with 10^−3^ dilution, while the RT-PCR could produce positive reaction with a 10^−2^ dilution (Figure 5c,d), indicating that theRT-RPA was approximately 10-fold more sensitive than the RT-PCR, based on the agarose gel electrophoresis assay. In addition, the sensitivity of the established RT-RPA assay was also evaluated by serial dilution of 1 μg total RNA from a CCGaV-infected apple with water, including 10^0^, 10^−1^, 10^−2^, 10^−3^ and 10^−4^. The results also indicated that the RT-RPA was approximately 10-fold more sensitive than the RT-PCR (Figure 5e,f). These results suggested that the established RT-RPA was a fast and simple method for CCGaV detection with high specificity and sensitivity.

### 2.4. Application of RT-RPA Assay for CCGaV Detection in Field-Collected Apple Plants

**Figure 6:**
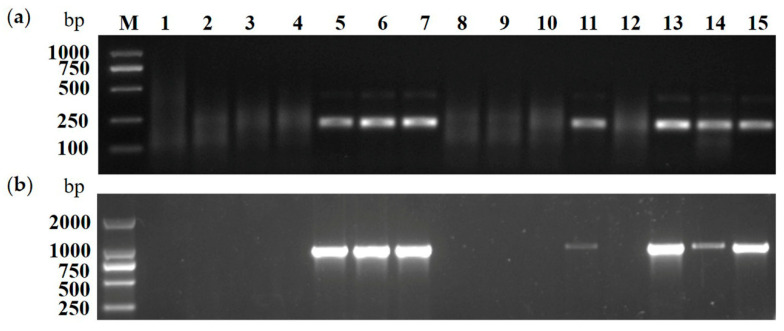
Detection of CCGaV infection in apple fruits from Shandong Province. (**a**) RT-RPA assay. (**b**) RT-PCR assay. Lane M, Trans2K DNA marker; lane 1, the NTC; lane 2, non CCGaV-infected apple plant; lane 3–15, apple samples from Shandong Province.

To evaluate the feasibility of the RT-RPA method for CCGaV detection in the field-collected samples, 76 apple samples with different virus-like symptoms (including bright stripes on apples, mosaic or mottled leaves and rusty skin) collected from Shandong Province (13), Xinjiang Province (57), Shaanxi Province (5) and Liaoning Province (1) were tested by RT-RPA and RT-PCR assays, respectively. The results showed that 7 out of the 13 samples from Weihai in Shandong Province were CCGaV-positive, per the RT-RPA and RT-PCR assays (Figure 6), while the other samples tested negative. These results demonstrated that the established RT-RPA assay was a reliable technique for CCGaV detection, and could be successfully applied in large-scale testing.

## 3. Discussion

Fruit trees are crops of high economic value and are widely cultivated around the world. The prevention and control of viral diseases is a concern and remains a great challenge during fruit production. Recently, the application of HTS has dramatically improved the detection of new viruses and viral isolates in fruit trees [12]. In the present study, we identified and characterized a new CCGaV-Weihai isolate in apple samples by detection of a virome through HTS, which was firstly reported in China. CCGaV was first identified in *Citrus sinensis* in 2018 in Italy, and was later found in apple trees in several countries [4,5,6]. Sequence alignment and phylogenetic analysis showed that CCGaV-Weihai was most closely related to CCGaV-H2799 from apples in the United States and was relatively distantly related to the CCGaV from *Citrus sinensis* in Italy, implying a possibly geographical and host differentiation of CCGaV isolates.

According to the reported survey of a global collection of apple trees in United States, 8 out of 22 samples from the USA, 3 out of 3 samples from Spain, 1 out of 1 sample from Australia, 1 out of 1 sample from Italy and 2 out of 2 samples from France were CCGaV-positive [5], which indicated that CCGaV is globally distributed in apples and prompted us to investigate the distribution of CCGaV in China. Through field surveys, we collected 76 apple samples from four major apple-producing provinces, and found that only the samples from Shandong Province were CCGaV-positive and the infection rate was 7 out of 13, which demonstrated that the infection rate of CCGaV was relatively high in some parts of China. The detailed geographic distribution and occurrence of CCGaV in China requires more samples data to clarify.

In addition, through detailed viral detection in the 13 apple fruits with obvious bright stripe symptom from Shandong Province, all samples were mix-infected by several different viruses. Among the six viruses and two viroids, the infection rate of ARWV1 was relatively low (23%) (Table S3). The infection rate of ACLSV (100%), ASPV (77%), ASGV (100%), ApNMV (100%), AHVd (100%) and ASSVd (100%) were very high (Table S3), while no studies have reported on the relationship between these viruses in combination with bright stripe symptoms on fruits. The CCGaV infection rate in Shandong Province (with bright stripe symptom) was 54% and the infection rate of samples from other provinces (with different virus-like symptoms but without bright stripe symptom) was 0, while the close correlation between the bright stripe symptoms of apple fruits and CCGaV infection still cannot be established. The pathogenicity of CCGaV infection in apple needs further research.

Apple trees can live for up to 50 years, and their productive and fertile period can last for several decades. Viruses are very common pathogens potentially affecting the growth of apple trees and the yield and quality of their fruits [1,2]. CCGaV is one of the pathogens that has been proposed to be putatively associated with concave gum-blind pocket disease in citrus, and it has been detected in apples in several regions and countries, which implies that the prevention of CCGaV needs to be improved in apple production. The detection of viruses and the use of certified virus-free propagation material is the most important strategy for viral disease management in fruit trees. The rapid, accurate and sensitive detection of viruses is a prerequisite for any such strategy. In the present study, we first established a detection method of CCGaV by RT-RPA. The developed RT-RPA assay could yield a detectable signal within 30 min (excluding the gel electrophoresis) at a constant temperature (38 °C), while RT-PCR required about 2–3 h and varying cycling temperatures. The specificity of RT-RPA for CCGaV detection was high and the presence of other common viruses or viroids (i.e., ApNMV, ACLSV, ASGV, ASPV, ARWV1, AHVd and ASSVd) did not interfere with CCGaV detection. Moreover, the sensitivity of the RT-RPA assay was about 10 times higher than that of conventional RT-PCR method, based on the agarose gel electrophoresis. Compared to RT-PCR, the shorter time, the single temperature with a simple heating device and the less stringent conditions makes it more suitable for large-scale sample detection.

## 4. Materials and Methods

### 4.1. Sample Collection and RNA Sequening

In October 2020, 13 apple fruits with bright stripe symptoms were collected from different apple trees grown in Shandong Province, China. In July 2021, 57 apple samples from Xinjiang Province, 5 from Shaanxi Province and 1 from Liaoning Province with viral symptoms such as mosaic or mottles leaves and rusty skin were collected. The fruit peels and apple leaves were taken and stored at −80 °C.

The fruit peels and apple leaves were used for total RNA extraction with the CTAB method (2% CTAB, 4% PVP, 100 mM Tris-HCl, 25 mM EDTA, 2 M NaCl, pH 8.0). The Ribo-Zero^TM^ rRNA removal kit (plant leaf) (Epicentre, Madison, WI, USA) was selected to deplete ribosomal RNA from the total RNA resulting in the ribo-depleted RNA sample, which was used to construct the cDNA library with a TruSeq RNA Sample Prep Kit v2 (Illumina, San Diego, CA, USA). Then, the library was subjected to high-throughput sequencing using the Illumina HiSeq 4000 platform with a paired-end 150 setup (Novogene, Beijing, China) (see Section 4.3). The raw reads from the HTS were initially processed by adapter trimming, quality trimming, and length trimming to obtain clean reads. To detect viruses in symptomatic apple fruits, the clean reads were mapped to the reference assembly genome of *Malus domestica* cv. Golden Delicious (NCBI GenBank assembly accession: GCA_002114115.1) for exclusion, while the unmapped reads were extracted for further analysis. The de novo assembly of contigs (see Section 4.3) were used as queries for BLAST against the NCBI databases (https://blast.ncbi.nlm.nih.gov/Blast.cgi, accessed on 30 August 2021).

### 4.2. Full-Length Genome Amplification and Sequencing

One µg of total RNA from apple peels, random primers, and M-MLV reverse transcriptase (Promega Corporation, Beijing, China) were used for the synthesis of first-strand cDNA. RT-PCR was performed to detect and amplify the viral genomic sequences. The RT-PCR products were purified with an AxyPrep DNA Gel Extraction Kit (Axygen Biotechnology Co., Silicon Valley, CA, USA) and then cloned into p-Topo-Blunt vector (Aidlab, Beijing, China). The recombinant clones were sequenced with universal primer pairs M13F/M13R via Sanger sequencing (TsingKe Biotech Co., Beijing, China). At least three independent clones were sequenced. The 5′- and 3′-terminal sequences of the genomic RNAs were confirmed through 5′-RACE and 3′-RACE using a SMARTer RACE cDNA Amplification Kit (Clontech, Mountain View, CA, USA) according to the manufacturer’s instructions. The specific primer pairs used in the RT-PCR experiments, the 5′ gene specific primers (GSPs) and the 3′ GSPs for the 5′-RACE and 3′-RACE were designed according to the assembled contigs and listed in Table S2.

### 4.3. Sequence Assembly and Bioinformatics Analyses of CCGaV

Sequences were analyzed and assembled with DNAMAN software, version 5.0 (Lynnon Biosoft, Quebec, QC, Canada) and then the assembled whole sequences were submitted to the GenBank database in NCBI using the WWW-based submission tool BankIt. The ClustalW method was applied to multiple sequence alignments, and MEGA 7.0 was used for phylogenetic tree construction using the maximum-likelihood method with the setting values of 1000 bootstrap replicates, Poisson model and complete deletion for gaps/missing data treatment options [21]. The sequence pair identities were calculated and aligned using MAFFT program (https://www.ebi.ac.uk/Tools/msa/mafft/, accessed on 30 August 2021), then the SDT software displayed the pairwise identity scores using a color-coded matrix [22]. The sequences used for comparison were obtained from the GenBank database.

### 4.4. RT-RPA Assay for CCGaV Detection

Two pairs of primers (Table S2) for RT-RPA were designed based on the genomic sequence of the CCGaV-Weihai isolate according to the guidelines for designing RPA primers, which recommend the length of primers be at least 30 nt and the amplicons no more than 500 bp. The RT-RPA assay was carried out following the instructions of the manufacturer of the TwistAmp Basic kit (TwistDX, Cambridge, UK). The 50 μL reaction volume contained 29.5 μL of rehydration buffer (containing recombinase, single-stranded binding protein and polymerase), 2.4 μL of each RPA-CCGaV-F/R primer (10 μM), 12.2 μL of nuclease-free water, 1.0 μL of cDNA and 2.5 μL of magnesium acetate (280 mM). The amplicons were purified using chloroform and then subjected to 13,000 rpm centrifugation for 3 min to pellet the protein. Five μL of supernatant were taken for analysis by 2% agarose gel electrophoresis. The NTC and healthy apple plant were used as negative controls in the RT-RPA assay. To optimize the reaction temperature and time, RPA reactions were performed from 36 °C to 39 °C and 10–50 min using the cDNA from CCGaV-infected apple plants. To assess the sensitivity, on the one hand, cDNA reverse-transcripted from 1 μg total RNA extracted from CCGaV-infected apple peels was diluted to 10^0^, 10^−1^, 10^−2^, 10^−3^ and 10^−4^ with water; on the other hand, 1 μg of total RNA was diluted to 10^0^, 10^−1^, 10^−2^, 10^−3^ and 10^−4^ with water and then reversed-transcripted into cDNA. The diluted templates were then used for the RPA assay. To evaluate the feasibility of the RT-RPA for CCGaV detection in the field, the field-collected apple plants were detected by RT-RPA and RT-PCR.

## 5. Conclusions

This study is the first report on the characterization of the CCGaV-Weihai isolate, offering preliminary investigation of local incidences in China and a novel isothermal amplification technique for the detection of CCGaV-infected apple trees. The full-length genomic sequences of CCGaV-Weihai RNA1 and RNA2 were determined to be 6674 nt and 2706 nt in length. Sequence alignment and phylogenetic analysis showed that CCGaV-Weihai was most closely related to CCGaV-H2799 from apples in the United States, and distantly related to CCGaV-CGW2 from *Citrus sinensis* in Italy, indicating a possible geographical and host differentiation of CCGaV isolates. Additionally, a reliable and sensitive RT-RPA assay was established for the rapid detection of CCGaV in apple plants, which is not interfered with by other common apple viruses and is about 10-fold more sensitive than the conventional RT-PCR method.

## Data Availability

Not applicable.

## Supplementary Materials

The following are available online at https://www.mdpi.com/article/10.3390/plants10112239/s1, Figure S1: RT-PCR detection of the putative viruses identified by HTS in the apple fruit samples. Lane M, Trans2K DNA marker; lane 1, apple chlorotic leaf spot virus (ACLSV); lane 2, apple stem pitting virus (ASPV); lane 3, apple stem grooving virus (ASGV); lane 4, apple necrotic mosaic virus (ApNMV); lane 5, apple rubbery wood virus 1 (ARWV1); lane 6, citrus concave gum-associated virus (CCGaV); lane 7, apple hammerhead viroid (AHVd); lane 8, apple scar skin viroid (ASSVd). Figure S2: Establishment of RT-RPA assay with primer pairs RPA-F1/R1 and RPA-F2/R2. Lane 1 and lane 4, non-template control; lane 2 and lane 5, non CCGaV-infected apple plant; lane 3 and lane 6, CCGaV-infected apple fruit. Table S1: Viral reads in diseased apples by analysis of high-throughput sequencing data. Table S2: Primers used in this study. Table S3: Detailed detection of six viruses and two viroids in 13 fruit samples from Shandong Province. Table S4: Amino acid sequences identities of CCGaV-Weihai and different CCGaV isolates from different countries and hosts. Table S5 Pairwise identity of CCGaV isolates.

## References

[1] Parish C.L. (1989). Apple decline: Characterization, cause and cure. Acta Hortic..

[2] Desvignes J.C., Boyé R. (1989). Different diseases caused by the chlorotic leaf spot virus on the fruit trees. Acta Hortic..

[3] Kuhn J.H., Adkins S., Alioto D., Alkhovsky S.V., Amarasinghe G.K., Anthony S.J., Avšič-Županc T., Ayllón M.A., Bahl J., Balkema-Buschmann A. (2020). 2020 taxonomic update for phylum *Negarnaviricota* (*Riboviria*: *Orthornavirae*), including the large orders *Bunyavirales* and *Mononegavirales*. Arch. Virol..

[4] Navarro B., Minutolo M., De Stradis A., Palmisano F., Alioto D., Di Serio F. (2017). The first phlebo-like virus infecting plants: A case study on the adaptation of negative-stranded RNA viruses to new hosts. Mol. Plant Pathol..

[5] Wright A.A., Szostek S.A., Beaver-Kanuya E., Harper S.J. (2018). Diversity of three bunya-like viruses infecting apple. Arch. Virol..

[6] Nickel O., Fajardo T.V.M., Candresse T. (2020). First report on detection of three bunya-like viruses in apples in Brazil. Plant Dis..

[7] Navarro B., Zicca S., Minutolo M., Saponari M., Alioto D., Di Serio F. (2018). A negative-stranded RNA virus infecting citrus trees: The second member of a new genus within the order *Bunyavirales*. Front. Microbiol..

[8] Xin M., Cao M., Liu W., Ren Y., Zhou X., Wang X. (2017). Two negative-strand RNA viruses identified in watermelon represent a novel clade in the order *Bunyavirales*. Front. Microbiol..

[9] Zhang S., Tian X., Navarro B., Di Serio F., Cao M. (2021). Watermelon crinkle leaf-associated virus 1 and watermelon crinkle leaf-associated virus 2 have a bipartite genome with molecular signatures typical of the members of the genus *Coguvirus* (family *Phenuiviridae*). Arch. Virol..

[10] Bertazzon N., Chitarra W., Angelini E., Nerva L. (2020). Two new putative plant viruses from wood metagenomics analysis of an Esca diseased vineyard. Plants.

[11] Chiapello M., Rodríguez-Romero J., Nerva L., Forgia M., Chitarra W., Ayllón M.A., Turina M. (2020). Putative new plant viruses associated with *Plasmopara viticola*-infected grapevine samples. Ann. Appl. Biol..

[12] Hou W., Li S., Massart S. (2020). Is there a “biological desert” with the discovery of new plant viruses? A retrospective analysis for new fruit tree viruses. Front. Microbiol..

[13] Kapoor S., Handa A., Sharma A. (2018). Prunus necrotic ringspot virus in peach-A bird’s eye view on detection and production of virus free plants. Int. J. Chem. Stud..

[14] Piepenburg O., Williams C.H., Stemple D.L., Armes N.A. (2006). DNA detection using recombination proteins. PLoS Biol..

[15] West S.C. (2003). Molecular views of recombination proteins and their control. Nat. Rev. Mol. Cell Biol..

[16] Londoño M.A., Harmon C.L., Polston J.E. (2016). Evaluation of recombinase polymerase amplification for detection of begomoviruses by plant diagnostic clinics. Virol. J..

[17] Mekuria T.A., Zhang S., Eastwell K.C. (2014). Rapid and sensitive detection of little cherry virus 2 using isothermal reverse transcription-recombinase polymerase amplification. J. Virol. Methods.

[18] Zhang S., Ravelonandro M., Russel P., McOwen N., Briard P., Bohannon S., Vrient A. (2014). Rapid diagnostic detection of plum pox virus in *Prunus* plants by isothermal AmplifyRP^®^ using reverse transcription-recombinase polymerase amplification. J. Virol. Methods.

[19] Kim N.Y., Lee H.J., Jeong R.D. (2019). A portable detection assay for apple stem pitting virus using reverse transcription-recombinase polymerase amplification. J. Virol. Methods.

[20] Jiao J., Kong K., Han J., Song S., Bai T., Song C., Wang M., Yan Z., Zhang H., Zhang R. (2020). Field detection of multiple RNA viruses/viroids in apple using a CRISPR/Cas12a-based visual assay. Plant Biotechnol. J..

[21] Sudhir K., Glen S., Koichiro T. (2016). MEGA7: Molecular evolutionary genetics analysis version 7.0 for bigger datasets. Mol. Biol. Evol..

[22] Muhire B.M., Varsani A., Martin D.P. (2014). SDT: A virus classification tool based on pairwise sequence alignment and identity calculation. PLoS ONE.

