# Supplementary material for: Characterization of an Isolate of Citrus Concave Gum-Associated Virus from Apples in China and Development of an RT-RPA Assay for the Rapid Detection of the Virus
Source: Plants (Basel). 2021 Oct 20;10(11):2239. doi: 10.3390/plants10112239 (PMC8621397; doi:10.3390/plants10112239)
Supplement: Supplementary file 1 [file plants-10-02239-s001.zip › Table S1 Viruses data.pdf]

Table S1 Viral reads in diseased apple by analysis of high throughput sequencing data

Viruses	Largest Contig Size (nt)	Viral Reads	% of Total Reads
apple stem pitting virus (ASPV)	2803	154284	0.2162
apple stem grooving virus (ASGV)	6511	195148	0.2735
apple chlorotic leaf spot virus (ACLSV)	3440	162461	0.2277
apple necrotic mosaic virus (ApNMV)			
RNA1	3362	334004	0.4682
RNA2	2680	372387	0.5220
RNA3	1952	958548	1.3435
Citrus concave gum-associated virus (CCGaV)			
RNA1	6661	22994	0.0322
RNA2	1373+1297	20879	0.0293
apple rubbery wood virus 1 (ARWV1)			
Segment L	7211	7801	0.0109
Segment M	1256	rare	-
Segment S	1042	3545	0.0050
apple hammerhead viroid (AHVd)	435	472439	0.6622
apple scar skin viroid (ASSVd)	328	100524	0.1409
