# Supplementary material for: Characterization of an Isolate of Citrus Concave Gum-Associated Virus from Apples in China and Development of an RT-RPA Assay for the Rapid Detection of the Virus
Source: Plants (Basel). 2021 Oct 20;10(11):2239. doi: 10.3390/plants10112239 (PMC8621397; doi:10.3390/plants10112239)
Supplement: Supplementary file 1 [file plants-10-02239-s001.zip › Table S2 CCGaV Primer list revised.pdf]

Table S2 Primers used in this study

Primers name	Sequence (5′-3′)	Locations in Genome	Length PCR Amplicon (bp)	Reference Sequence
**CCGaV-RNA1**				
RNA1-F1	CACATGTGTACTCAGCATCCTG	296-317	1537	MZ926713
RNA1-R1	GCTAAGAGTGCTAAGGAAGTGG	1832-1811		
RNA1-F2	CACTCTCCTAGTCCTGATCTAGTC	1689-1712	1674	MZ926713
RNA1-R2	CAGGACAAGGTTAGCAACAAC	3362-3342		
RNA1-F3	TGATGACTTAG CCCTGCTAG	3275-3294	1527	MZ926713
RNA1-R3	CCATACAGATTTTGTGAGAGG	4801-4781		
RNA1-F4	ATCTTATCACCTCTGGCAAC	4762-4781	1445	MZ926713
RNA1-R4	CGTGTTGATTGTGTCTCCATC	6206-6186		
RNA1-3RACE	GATTACGCCAAGCTTCGCTTCATCTGCAGTGTGATCTTC	6041-6064	634	MZ926713
RNA1-5RACE-1	GCAGTTACAGGATGCTGAGTAC	324-303	324	MZ926713
RNA1-5RACE-2	GTGGCCGGGTATGTATACCAGTTCC	663-639	663	MZ926713
**CCGaV-RNA2**				
RNA2-F1	CTCGAGACTATAGACATCATGTTG	35-58	1286	MZ926714
RNA2-R1	CAGAAGATATGATACAAGATTCATGG	1320-1295		
RNA2-F2	TTACTGCTGATGACGTCTC	1149-1167	1482	MZ926714
RNA2-R2	GGCTGACAAGATAAACAACCC	2630-2610		
RNA2-5RACE	GATTACGCCAAGCTTGTTGTGGTAACTTAGACTGAGGCAGAGC	407-380	407	MZ926714
RNA2-3RACE	CTTGCCCGTCATCACCATCTCTAG	2425-2448	282	MZ926714
**CCGaV-RPA**				
CCGaV-RPA-F1	GGACCACTCTTTACAAAGTGCTCAATGCTAGGTTC	5001-5035	198	MZ926713
CCGaV-RPA-R1	GATCCACAAAGGCATACACATTCTGATTAGGTGC	5198-5165		
CCGaV-RPA-F2	GATAGATCTAGTTCCAAGTGAGACAACATGTGTG	4947-4980	175	MZ926713
CCGaV-RPA-R2	CCTGCAAGTTATTAGAAGTGGGGAGAATAGGACC	5121-5088		
**Virus Detection**				
ACLSV-5F	TCTGCAAGAGAATTTCAGTT	6720-6739	823	KC935956.1
ACLSV-3R	GTCTACAGGCTATTTATTATAAG	7542-7520		
ASPV-5F	CTCTTGAACCAGCTGATGGCC	9019-9039	264	NC_003462.2

ASPV-3R	ATAGCCGCCCCGGTTAGGTT	9282-9263		
ASGV-5F	CCCGCTGTTGGATTTGATACACCTC	5871-5895	524	NC_001749.2
ASGV-3R	CTGCAAGACCGCGACCAAGTTT	6394-6373		
ApNMV-5F	CAACCCGAGTTCATCCAG	-	507	Contigs from HTS in this study
ApNMV-3R	AGGTTTCAACTGCGTCTTG	-		
ARWV1-5F	CACGAGTCATTTGGCTGTC	-	435	Contigs from HTS in this study
ARWV1-3R	GTGTTAGCAAGGCAACTTTG	-		
CCGaV-5F	TGCCCATCCTTCTAACCTG	2731-2749	954	MZ926713
CCGaV-3R	TTCCCATACAGTTTGCCG	3684-3667		
ASSVd-5F	GTCGACGAAGGCCGGTGAGAAA	87-108	329	NC_001340.1
ASSVd-3R	GTCGACGACGACAGGTGAGTTCC	92-70		
AHVd-5F	TGCCGAAACAGAGGTTGG	-	232	Contigs from HTS in this study
AHVd-3R	CGTTCCAAGGACGAAACC	-		
