# Supplementary material for: Characterization of an Isolate of Citrus Concave Gum-Associated Virus from Apples in China and Development of an RT-RPA Assay for the Rapid Detection of the Virus
Source: Plants (Basel). 2021 Oct 20;10(11):2239. doi: 10.3390/plants10112239 (PMC8621397; doi:10.3390/plants10112239)
Supplement: Supplementary file 1 [file plants-10-02239-s001.zip › Table S4 CCGaV different isolates lists.pdf]

Table S4: Amino acid sequences identities of CCGaV-Weihai and different CCGaV isolates from different countries and hosts.

			GenBank accession no.			Identifies (%)		
**Isolate**	**Host**	**Country**	**RdRp**	**MP**	**CP**	**RdRp**	**MP**	**CP**
CCGaV-Gala	*Malus domestica*	Brazil	QDK54398	QDK54400	QDK54401	99.82	99.51	99.14
CCGaV-Mishima	*Malus domestica*	Brazil	QDK54399	QDK54402	QDK54403	99.77	99.51	99.43
CCGaV-AC1	*Malus domestica*	USA	AXR98526	AXR98528	AXR98527	98.81	99.02	99.14
CCGaV-LR3	*Citrus sinensis*	Italy	AYN78565	AYN78566	AYN78567	97.39	96.81	98.29
CCGaV-CGW2	*Citrus sinensis*	Italy	YP_009422199	YP_009407930	YP_009407931	97.30	97.30	98.29
CCGaV-H2799	*Malus domestica*	USA	QSC42549	---	---	99.91	---	---
CCGaV-FT159	*Malus sp.*	USA	QTH26260	---	---	98.99	---	---
