# Supplementary figures and images for: Characterization of an Isolate of Citrus Concave Gum-Associated Virus from Apples in China and Development of an RT-RPA Assay for the Rapid Detection of the Virus
Source: Plants (Basel). 2021 Oct 20;10(11):2239. doi: 10.3390/plants10112239 (PMC8621397; doi:10.3390/plants10112239)

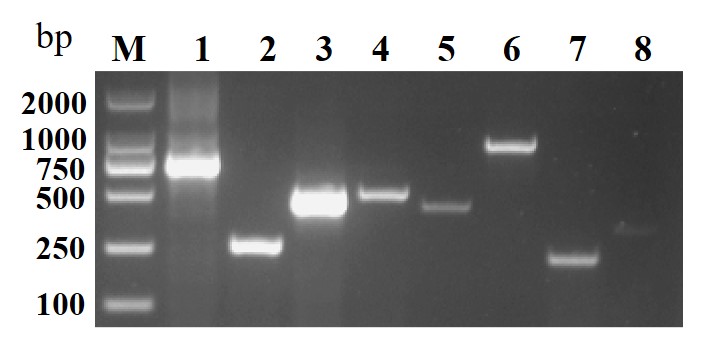

Supplement: Supplementary file 1 [file plants-10-02239-s001.zip › Figure S1 virus detection-.jpg]

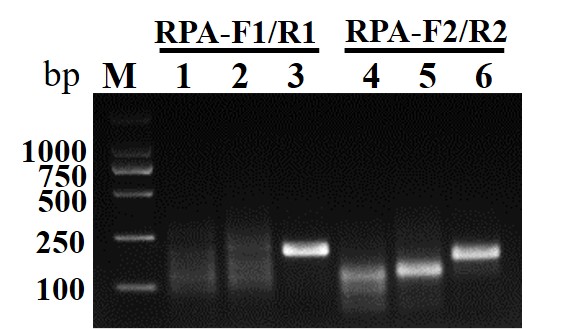

Supplement: Supplementary file 1 [file plants-10-02239-s001.zip › Figure S2 CCGaV isolates.jpg]
