# Supplementary material for: Characterization of an Isolate of Citrus Concave Gum-Associated Virus from Apples in China and Development of an RT-RPA Assay for the Rapid Detection of the Virus
Source: Plants (Basel). 2021 Oct 20;10(11):2239. doi: 10.3390/plants10112239 (PMC8621397; doi:10.3390/plants10112239)
Supplement: Supplementary file 1 [file Table S3 Detailed detection of six viruses and two viroids in 13 fruits samples from Shandong Province.pdf › Table S3 Detailed detection of six viruses and two viroids in 13 fruits samples from Shandong Province.pdf]

Table S3 Detailed detection of six viruses and two viroids in 13 fruits samples from Shandong Province

Sample No. / Viruses	1	2	3	4	5	6	7	8	9	10	11	12	13
**ACLSV**	+	+	+	+	+	+	+	+	+	+	+	+	+
**ASPV**	+	+	-	+	-	+	+	+	-	+	+	+	+
**ASGV**	+	+	+	+	+	+	+	+	+	+	+	+	+
**ApNMV**	+	+	+	+	+	+	+	+	+	+	+	+	+
**ARWV1**	+	-	-	-	-	+	+	-	-	-	-	-	-
**CCGaV**	-	-	+	+	+	-	-	-	+	-	+	+	+
**AHVd**	+	+	+	+	+	+	+	+	+	+	+	+	+
**ASSVd**	+	+	+	+	+	+	+	+	+	+	+	+	+
